# Potential Health Benefits of Bee Bread from Stingless Bees on Reproductive Health: A Review

**DOI:** 10.3390/ijms27125511

**Published:** 2026-06-18

**Authors:** Nurul Ain Kamar Bashah, Adila A. Hamid, Farah Hanan Fathihah Jaffar, Mohd Zulkifli Mustafa, Siti Hajar Adam, Siti Sarah Mohamad Zaid, Wan Iryani Wan Ismail, Muhammad Adib Dwi Tamma Putra, Mohd Helmy Mokhtar

**Affiliations:** 1 Department of Physiology, Faculty of Medicine, Universiti Kebangsaan Malaysia, Cheras, Kuala Lumpur 56000, Malaysia; nurulain_0917@yahoo.com (N.A.K.B.); adilahamid@ukm.edu.my (A.A.H.); farahhanan@ukm.edu.my (F.H.F.J.); 2 Department of Neuroscience, School of Medical Sciences, Universiti Sains Malaysia, Kubang Kerian, Kota Bharu 16150, Malaysia; zulkifli.mustafa@usm.my; 3 Preclinical Department, Faculty of Medicine & Defence Health, Universiti Pertahanan Nasional Malaysia, Kuala Lumpur 57000, Malaysia; siti.hajar@upnm.edu.my; 4 Department of Environment, Faculty of Forestry and Environment, Universiti Putra Malaysia, Serdang 43400, Malaysia; mz_sarah@upm.edu.my; 5 Cell Signaling and Biotechnology Research Group (CeSBTech), Faculty of Science and Marine Environment, Universiti Malaysia Terengganu, Kuala Terengganu 21030, Malaysia; waniryani@umt.edu.my; 6 Department of Physical Medicine and Rehabilitation, Faculty of Medicine, University of Sriwijaya Palembang, Kota Palembang 30128, Indonesia; adibdwi@unsri.ac.id; 7 Doctoral Program, AMA University, Quezon City 1106, Philippines

**Keywords:** bee bread, reproductive health, spermatogenesis, ovarian function, antioxidant, steroidogenesis

## Abstract

Bees are social insects belonging to the Apidae family, which includes stingless bees, honeybees, and related groups. Their ability to produce various products, such as bee bread, bee pollen, propolis, beeswax, and royal jelly, has attracted scientific interest due to their nutritional composition, biological activities, and potential therapeutic value. Bee bread is a fermented mixture of pollen, honey, and salivary bee enzymes, rich in bioactive compounds with potential benefits for reproductive health and other biological activities. However, bee bread remains one of the least explored bee products in relation to reproductive health. This narrative review summarises the potential health benefits of bee bread from stingless bees for male and female reproductive function. Evidence from animal studies shows that bee bread has promising effects on reproductive function, possibly through its antioxidant properties, support of spermatogenesis and steroidogenesis, improvement of pregnancy outcomes, enhancement of ovarian function, regulation of metabolism, and modulation of inflammatory activity. Findings from animal studies suggest potential benefits for both male and female reproductive health. However, well-designed randomised controlled trials are needed to evaluate its efficacy, optimal dosage, safety profile, and long-term effects on reproductive outcomes in both males and females.

## 1. Introduction

Bees are social insects that form large groups and belong to the Apidae family. This family includes stingless bees, honeybees and other groups. Stingless bees are a diverse group within Hymenoptera, Apidae, Meliponini, comprising 600 species and subspecies across 60 groups [1]. They play an important role in pollination, similar to *Apis mellifera* [2]. Stingless bees show significantly greater biological activity due to their species diversity and wide geographical distribution in Southeast Asia, Australia, Africa, and South America [3,4]. This extensive biological and geographical diversity directly influences the complex phytochemical composition of bee bread, which forms the basis of its significant therapeutic effects on reproductive health.

Bee bread, also known as fermented pollen, pot-pollen, stored pollen, or ambrosia [5,6], is a bee by-product formed through natural fermentation in the beehive [5]. Bee-collected pollen is mixed with saliva and honey, then packed into honeycomb cells and stored in the cerumen pot by indigenous microbes [7]. These microbes are essential for protecting bee colonies against pathogens and for providing nutrients necessary for bee growth [8]. After two weeks, bee bread forms at 35 °C to 36 °C with moisture, various enzyme activities from glandular secretions, and microorganisms, including bacteria and yeasts [9]. Bee bread is used by young and worker bees to produce royal jelly and to feed larvae [5]. The nutritional and bioactive compounds in bee bread are promising in terms of biological activity, which depends on the geographical and botanical origin of the pollen, the microbiota present in the cell, the chemical composition of bee species such as proteins, lipids, polyphenols, vitamins, and amino acids, as well as climatic conditions, soil type, beekeeping practices, and storage treatments in commercial production [9,10,11,12]. Such variations may contribute to discrepancies in reproductive outcomes observed among studies, highlighting the need to consider the source and composition of bee bread when assessing its efficacy.

The process of making bee bread begins with plant pollen collected by stingless bees. For example, *Heterotrigona itama* is attracted to foraging plants, especially white and cream-coloured flowers, closest to their hive [13,14], due to the high sugar concentration in the nectar [15]. During foraging, forager bees collect nectar and store it in their stomachs, which contain honey, while their bodies become covered in pollen dust. Stingless bees collect pollen from underutilised fruits, trees, ornamental plants, herbs, shrubs, lianas, and epiphytes [14,16] using their salivary enzymes, amylase and glucosidase, and honey [17,18] to moisten, agglutinate, and store the pollen in a “pollen basket” on their hind legs [19]. Forager bees transport the collected pollen and nectar back to the hive. Worker bees pack the pollen inside a cerumen pot for stingless bees or honeycomb cells for honeybees, both made from beeswax and resin, if the pollen is not harvested. The filled pollen pots are sealed to prevent water loss from the bee bread [20,21]. The transformation of pollen into bee bread occurs during lactic acid fermentation [22]. Fermentation improves the digestibility and bioavailability of bee bread by degrading the outer pollen layer [23,24]. A recent study explored the role of bee bread from nine colonies in three ecosystems (forest, forest–urban, and urban–farmland). The results showed that bee bread is associated with microbial communities in physicochemical characteristics and microbial composition, which contribute to its nutritional quality and to bee gut microbes [25].

Bee bread has been shown to possess therapeutic properties, including anti-inflammatory, antioxidant, antimicrobial, antitumour, and antihypertensive activities [26,27]. Various therapies are available to reduce inflammation, including adherence to an anti-inflammatory diet such as bee bread, which serves as a preventive measure. Flavonoids exert anti-inflammatory effects through multiple mechanisms, including inhibition of regulatory enzymes and transcription factors that control inflammatory mediators. They also have potent antioxidant properties, scavenging free radicals to reduce their harmful effects. Consequently, anti-inflammatory diets significantly reduce the risk of developing and progressing chronic, non-communicable diseases [28].

Reproductive health is influenced by hormonal balance, oxidative stress, and metabolic activity. Disruptions in these processes including oxidative stress, chronic inflammation, metabolic dysfunction, and endocrine dysregulation contribute to infertility and reproductive disorders such as polycystic ovary syndrome, endometriosis, testicular dysfunction, and impaired spermatogenesis. Bee bread has attracted attention for its potential to support reproductive function, mainly through its role in enhancing spermatogenesis and oogenesis, modulating reproductive hormones, and reducing oxidative damage in reproductive organs in animal studies [29,30,31,32,33].

**Figure 1:**
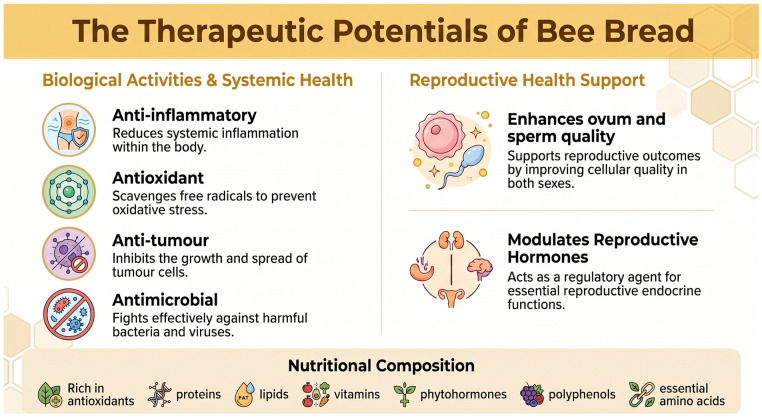
Summary of the therapeutic potentials of bee bread, highlighting its antioxidant, anti-inflammatory, antimicrobial, and anti-tumour properties, as well as its supportive roles in reproductive health, including improved sperm and ovum quality, and modulation of reproductive hormones.

Bee bread contains higher levels of bioactive components that can influence reproductive physiology, including antioxidants, proteins, lipids, phytohormones, polyphenols, vitamins, and essential amino acids [34]. These compounds have been investigated in reproductive research for their potential to improve sperm quality [29], reduce testicular dysfunction [30], improve pregnancy outcomes [31,35], enhance ovarian function [32], regulate sex hormones, and reduce oxidative and inflammatory damage in reproductive tissues [33], making bee bread a potential reproductive-supportive nutraceutical. It should be noted that most of the evidence regarding the reproductive benefits of bee bread originates from preclinical studies, and further clinical investigations are required to confirm its efficacy in humans. This review therefore examines the available evidence on the impact of bee bread on both male and female reproductive health, focusing on experimental findings. Figure 1 presents a summary of the therapeutic potentials of bee bread.

## 2. Methods

This article is a structured narrative review that comprehensively summarises the beneficial effects of bee bread on the reproductive system. Relevant peer-reviewed articles were identified through targeted searches of the PubMed/MEDLINE and Scopus databases. The literature search included articles published up to 2026. Key search terms and combinations included ‘stingless bee’, ‘bee bread’, ‘male reproduction’, and ‘female reproduction’. Due to the narrative design of this review, formal systematic methodologies such as a PRISMA flow diagram, independent duplicate screening, risk-of-bias assessments, or quantitative data synthesis were not employed.

## 3. Nutritional and Physicochemical Properties of Bee Bread from Stingless Bees

Bee bread flavours vary among species. Sweet bee bread is produced by *Tetragonula angustula*, *Ptilotrigona*, *Frieseomelitta doederlini*, and *Frieseomelitta varia*, while *Melipona* and *Scaptotrigona* produce bitter bee bread [21,36]. Studies by Kostić et al. (2015) and McFrederick et al. (2012) indicate that bee bread is an ideal food because of its nutritional value, being rich in carbohydrates, proteins, lipids, fatty acids, minerals, vitamins, phenolic compounds, and essential amino acids [26,37,38,39].

The addition of nectar during the transformation of pollen into bee bread increases its carbohydrate content [40,41]. In *H. itama*, the carbohydrate content of bee bread has been reported to range from 22.36% to 58.73%, with specific values of 55.1% and 58.2% found in different studies [13,42,43]. Furthermore, bee bread contains higher crude ash and lower cellulose [44], as well as reducing sugars such as fructose, glucose, sucrose, mannitol, and fibre [45,46,47].

Bee bread of *H. itama* contains lipid levels ranging from 2.17% to 4.80% and 5.3% in different studies [13,43]. Crude fibre levels have been reported at 9.30% in *Meliponini seminigra* and 13.65% in *M. interrupta* [48]. In addition, bee bread has a water activity between 0.60 and 0.90 and is more acidic than bee pollen. For example, water activity in bee bread from *H. itama* ranges from 0.73 to 0.85 [13]. Studies on the pH of European bee pollen show that the pH decreases from 4.7 to 3.97 after transformation into bee bread [49]. Similarly, De Jesus Oliveira et al. (2020) reported that the lowest pH in bee bread of *Melipona scutellaris* is 3.28 [21]. Moisture content has been reported at 8.10% and between 11.09% and 12.51% [13,42], while crude ash content in *H. itama* ranges from 1.70% to 2.54% [13,42].

Bee bread has a higher moisture content due to water absorbed from the environment and the addition of bee saliva and honey. The protein content in *H. itama* has been reported as 47.4%, 17.22% to 18.37%, and 22.6% [13,42,43], indicating that bee bread is becoming the main protein source for bee development because of its high protein content. Bee bread contains all the essential amino acids [38,50,51] and bioactive compounds such as phenolics, flavonoids, carotenoids, unsaturated fatty acids, enzymes, and probiotics [4]. Variability in composition between studies, resulting from differences in botanical sources, bee species, and analytical procedures, may affect the reliability and comparability of the findings. This heterogeneity should therefore be considered when evaluating any biological or therapeutic effects of bee bread. Table 1 presents the nutritional and physicochemical properties of bee bread from various stingless bee species.

Bee bread is continually enriched with phenolic compounds, including flavonoids and phenolic acids, which are responsible for its biological activities such as antioxidant, antibacterial, and anti-inflammatory effects [52]. The complex matrix of bee bread is reflected in the range of analytical techniques used to identify and confirm these compounds, such as LC-MS, HPLC-UV/DAD, LC-ESI-IT-MS/MS, and column chromatography with NMR [53].

In *H. itama* from Malaysia, bee bread contains key phenolic acids that contribute to its biological activity such as caffeic acid and ferulic acid, along with flavonoids including kaempferol, apigenin, and isorhamnetin. Isorhamnetin showed the highest mass spectral intensity and was the most prevalent compound detected in the sample. Kaempferol and apigenin followed, both flavonoids known for their antioxidant and anti-inflammatory properties [54]. Additionally, phenolic acids such as ferulic acid and caffeic acid were identified, further enhancing the overall antioxidant capacity of the bee bread [30,55]. These compounds are known for strong free radical scavenging and anti-inflammatory activities, providing significant antioxidant potential in Malaysian stingless bee bread [56].

In contrast, *Melipona subnitida* from Brazil exhibits a broader range of flavonoids, including naringenin, isorhamnetin derivatives, tricetin, selagin, and methoxyherbacetin, indicating a more chemically diverse profile influenced by *Fabaceae* and *Scrophulariaceae* plant sources [57]. Bee bread from Venezuelan *Frieseomelitta* species is dominated by classical flavonoids such as quercetin, kaempferol, and luteolin, which are commonly associated with cardiovascular protection and antimicrobial activity [11,58]. Similarly, several *Melipona* species such as *M. compressipes*, *M. eburnean*, *M. favosa*, *M. fulva*, and *M. lateralis kangarumensis,* consistently contain flavonoids such as kaempferol, luteolin, quercetin, and methoxykaempferol. This indicates a conserved biosynthetic preference within the genus for flavonoid-rich bee bread [11]. In addition, some species also produce unique compounds such as genkwanin, further enhancing phytochemical diversity [59].

More complex metabolite profiles are observed in *M. rufiventris*, which contains not only flavonoids such as quercetin, luteolin, and isorhamnetin but also phenolic acid derivatives like p-hydroxycinnamic acid and glycosylated flavonoids, indicating advanced biotransformation during bee bread maturation [47]. Meanwhile, *Scaptotrigona affinis postica* exhibits one of the most chemically diverse profiles, including phenolic acids (protocatechuic acid, ellagic acid), flavonoid glycosides (quercetin- and kaempferol-derived compounds), and procyanidin dimers, suggesting strong antioxidant and potential anti-cancer properties [60,61].

Similarly, *M. fasciculata* bee bread contains both phenolic compounds and lipid-related molecules such as linolenic and linoleic acids, indicating that bee bread is a source of both polyphenols and bioactive fatty acids [62]. *Tetragonula biroi* from the Philippines, by contrast, shows a simpler yet still bioactive profile dominated by rutin, quercetin-3-O-glucoside, and quercetin, compounds widely recognised for their vascular and antioxidant benefits [45].

Thus, stingless bee bread is a chemically complex functional food rich in polyphenols, flavonoids, phenolic acids, and, in some cases, lipid derivatives. Variation in its composition reflects differences in floral sources, bee species-specific enzymatic processing, and regional biodiversity. These factors collectively determine the pharmacological potential of bee bread and support its use as a natural antioxidant, antimicrobial, and nutraceutical product.

## 4. Potential Health Benefits of Bee Bread from Stingless Bees on Reproductive System

Bee bread has emerged as a promising functional apitherapeutic with multiple roles in reproductive health, largely due to its rich bioactive composition and pleiotropic molecular actions. Animal studies have shown that bee bread may benefit in reproductive health through its antioxidant [29,30,63], anti-inflammatory [29,30,32,33,63], anti-apoptotic [29,30,32,63], metabolic modulatory [32,63,64], upregulation of steroidogenesis [29,33,63,65], improvement of pregnancy outcomes [31,35,66] and autophagy-activating properties [32].

### 4.1. Effects of Bee Bread on Male Reproductive Health

#### 4.1.1. Spermatogenesis and Sperm Quality

Spermatogenesis is a continuous, highly organised process in which spermatogonia undergo mitotic and meiotic divisions and complex cytological transformations, resulting in the formation of sperm throughout the adult life of a male [67,68,69]. The sequence of events from the disappearance of a given cell association to its reappearance in a specific area of the seminiferous epithelium constitutes the cycle of the seminiferous epithelium [70,71].

Research into the potential health benefits of bee bread supplementation for male reproductive organs and sperm parameters has attracted increasing attention. A study by Zakaria & Haron (2018) showed the effects of bee bread from the stingless bee *H. itama* on these parameters [72]. In this study, rats were divided into treated and control groups, each containing six rats. The control group received 1 mL of distilled water, while the treated group was given 0.5 g/kg/body weight of bee bread diluted in 1 mL of distilled water for 28 days. Evaluation of sperm parameters from the cauda epididymis showed that bee bread supplementation significantly increased prostate gland weight and improved sperm quality. Testicular androgens are important for the prostate’s growth and function throughout life, particularly in supporting cell growth and survival within the gland [73,74]. Accordingly, androgens have remained a central focus in prostate research, with broad consensus that they are indispensable for normal prostatic growth, development, and the preservation of tissue homeostasis [75,76]. During the pre-pubertal and pubertal stages, the intraprostatic conversion of testosterone to dihydrotestosterone is widely regarded as a mechanism driving prostate enlargement to its mature size [73]. The enlargement of the prostate gland may suggest a stimulatory effect of bee bread on androgen-responsive accessory sex organs, which is commonly associated with enhanced androgen activity [75]. The findings indicate that bee bread may exert androgenic effects on accessory sex glands, thereby improving spermatogenesis and overall reproductive function [65,72,77]. However, the potential effects on androgen-responsive tissues, including the prostate, remain uncertain, and the available evidence is limited. Possible risks, limitations, and the lack of clinical validation should be considered when interpreting these findings. In addition, improved sperm motility and viability are essential factors in male fertility, as they directly influence the ability to achieve successful fertilisation [78]. Therefore, these findings suggest bee bread may have protective and stimulatory effects on the male reproductive system, attributable to its high concentrations of antioxidants, vitamins, amino acids, and bioactive compounds [34,79].

#### 4.1.2. Hormone Regulation and Steroidogenesis

Bee bread has been reported to have a protective effect against obesity-associated reproductive dysfunction. In a study in which male rats were fed a high-fat diet (HFD) to induce obesity and assess its impact on reproductive parameters, the levels of leptin, malondialdehyde, and sperm DNA fragmentation increased significantly in the HFD group [63]. This indicates metabolic imbalance, increased oxidative stress, and impaired sperm integrity [80]. Additionally, there were decreases in the testicular mRNA transcript levels of androgen receptor, luteinising hormone receptor, steroidogenic acute regulatory protein, cytochrome P450 enzyme, 3β-hydroxysteroid dehydrogenase (HSD), and 17β-HSD in the testes of the HFD group [63]. These genes are crucial for testosterone synthesis and for maintaining normal spermatogenesis [81,82,83]. Their downregulation in the high-fat diet group indicates impaired steroidogenesis and reduced testicular function [84]. Supplementation with *H. itama* bee bread at a dosage of 0.5 g/kg body weight substantially alleviated these adverse effects. Administration of this bee bread decreased circulating leptin levels and increased adiponectin levels, indicating improved metabolic and adipose tissue regulation. It also improved sperm parameters, reduced sperm DNA fragmentation, and increased the expression of steroidogenic genes and proteins in obese rats [63]. The study concluded that bee bread may be considered a potential supplement to protect against infertility in overweight and obese men.

In addition to its antioxidant and metabolic benefits, bee bread (*H. itama*) has also been shown to improve testicular development and testosterone levels in Sprague Dawley rats. Zakaria et al. (2023) investigated the effects of bee bread supplementation on testicular histomorphology and testosterone concentration in adult male Sprague Dawley rats [65]. In this study, twenty-four adult male Sprague Dawley rats were divided into a control group and three treatment groups, which received 1 g, 2 g, and 3 g of bee bread per kilogram of body weight, respectively. Bee bread was administered daily for 28 days. The study found that rats in the 2 g bee bread per kilogram body weight group had a greater seminiferous tubular diameter and higher seminiferous epithelial height compared to the control group. These structural enhancements indicate increased spermatogenic activity and more vigorous growth of the germinal epithelium in the testes [65,72,85]. Furthermore, the expansion of seminiferous tubules and the increased thickness of the seminiferous epithelium are typically associated with active spermatogenesis and enhanced reproductive capability [86,87]. Testosterone levels in the treated groups were significantly higher, and bee bread supplementation improved testicular cell development and testosterone levels in male Sprague Dawley rats. This suggests that the function of Leydig cells and the process of steroidogenesis are positively influenced by bee bread [65,72]. The enhancement of testosterone production is crucial, as this hormone is essential for maintaining spermatogenesis, secondary sexual characteristics, and the overall health of the male reproductive system [88].

Furthermore, a study on the inhibitory effects of bee bread extracts from *Lepidotrigona flavibasis* (BBLF) on testosterone propionate (TP)-induced benign prostatic hyperplasia (BPH) in mice showed that animals treated with TP and BBLF (300 mg/kg) had a significant reduction in prostate index compared with the TP-only group, indicating attenuation of prostate enlargement [33]. In addition, histopathological analysis indicated partial restoration of prostatic architecture, characterised by reduced thickness and elongation of the glandular epithelium, suggesting decreased abnormal epithelial proliferation [33]. Furthermore, serum concentrations of oestradiol, interleukin-1β (IL-1β), and interleukin-6 (IL-6) were markedly reduced in the TP + BBLF group compared with the TP group. These data suggest that BBLF has anti-inflammatory properties and may affect hormone-related pathways involved in the aetiology of BPH [26,62]. Increased oestradiol and pro-inflammatory cytokines are recognised factors in prostatic hyperplasia, promoting cellular proliferation and inflammatory signalling [89,90]. The study further indicates that BBLF exhibits considerable inhibitory effects on TP-induced BPH, presumably due to its antiproliferative and anti-inflammatory properties. These findings highlight the potential of bee bread extracts as a natural medicinal alternative for the treatment of prostate hyperplasia. Although the findings are promising in experimental models, they should be interpreted as preliminary evidence, and further studies are needed to confirm safety, efficacy, and mechanistic pathways in reproductive systems.

#### 4.1.3. Oxidative Stress, Inflammation, and Apoptosis

Apoptosis, also known as programmed cell death, is a cellular process vital for the normal development and homeostasis of all multicellular organisms [91,92,93]. This mechanism involves the elimination of damaged, infected, or potentially neoplastic cells. However, both insufficient and excessive apoptotic activity can lead to detrimental biological effects [94]. Mitochondria are the primary site for the generation of reactive oxygen species (ROS), including superoxide anion, hydroxyl radical, singlet oxygen, and hydrogen peroxide [95]. It is estimated that 1% to 4% of oxygen interacting with the mitochondrial respiratory chain results in the formation of superoxide radicals. Additional sources of ROS include radiation, cytotoxic chemicals, and pharmaceutical agents. Elevated oxidative stress can induce cell death through either necrosis or apoptosis [96,97].

A study on HFD-induced obese rats treated with bee bread demonstrated the potential protective effect of bee bread (*H. itama*) on testicular oxidative stress, inflammation, apoptosis, and lactate transport in the testes of obese rats [29,30]. Obesity is generally known to disrupt redox balance, cause chronic inflammation, and impair sperm formation [98]. The findings show that HFD exposure decreases mRNA expression of antioxidant genes, including nuclear factor erythroid 2–related factor 2 (*Nrf2*), superoxide dismutase (SOD), catalase (CAT), and glutathione peroxidase (GPx), indicating compromised antioxidant defence. In addition, mRNA levels of glucose transporters (Glut1 and Glut3), monocarboxylate transporters (Mct2 and Mct4), and lactate dehydrogenase type C (Ldhc) also decreased, suggesting impaired metabolic support for spermatogenesis. Simultaneously, HFD-induced obesity significantly increased pro-inflammatory markers, including nuclear factor kappa B (NF-κB), tumour necrosis factor-alpha (TNF-α), inducible nitric oxide synthase (iNOS), and interleukin-1 beta (Il-1β), as well as upregulating the expression of pro-apoptotic genes such as p53, *Bax*, the *Bax/Bcl-2* ratio, and caspases (Caspase-8, -9, and -3). This may be due to the presence of phenolic substances with documented anti-inflammatory actions, such as caffeic acid, gallic acid, trans-ferulic acid, trans-3-hydroxycinnamic acid, and 2-hydroxycinnamic acid. These molecular changes collectively indicate increased inflammation and activation of apoptotic pathways, resulting in testicular damage [99].

Treatment with bee bread significantly increased the expression and activity of antioxidant enzymes, restoring redox equilibrium. It also inhibited the expression of pro-inflammatory and pro-apoptotic genes, leading to reduced cellular stress and improved testicular health. Bee bread further enhanced the immunoexpression of proliferating cell nuclear antigen (PCNA), indicating improved cell growth and spermatogenic activity. The treatment also upregulated genes essential for lactate transport and metabolism, which are important for providing energy to developing germ cells. These findings demonstrate that bee bread provides several protective benefits to the testes of obese rats on a high-fat diet by reducing oxidative stress, inflammation, apoptosis, and metabolic dysfunction. Table 2 presents an overview of the biological activities and underlying mechanisms that support the potential benefits of bee bread in male reproductive health.

### 4.2. Effects of Bee Bread on Female Reproductive Health

#### 4.2.1. Pregnancy Outcomes and Stress Modulation

In animal studies, stress has been recognised as a potential factor influencing adverse pregnancy outcomes. Neuroendocrine systems typically react to acute stress, whether psychosocial or environmental, enabling the organism to adapt and respond effectively to changing conditions [100]. However, when stress becomes chronic or excessive, these regulatory mechanisms may be overwhelmed, and the stress response itself can contribute to pathological conditions [101]. Under stressful circumstances, the endocrine, nervous, and immune systems interact to maintain homeostasis. Prolonged stress can disrupt this balance, leading to maladaptive responses and long-term health consequences. Growing evidence links chronic stress exposure to negative effects on pregnancy and neonatal health [102,103,104].

Bee bread has shown significant promise in improving pregnancy outcomes and reducing physiological stress responses, particularly under challenging environmental conditions. Recent data suggest that it may naturally modulate stress hormones, potentially benefiting both mother and foetus in experimental models. Studies on pregnant Sprague Dawley rats exposed to heat stress found that bee bread (*H. itama*) supplementation had protective and restorative effects against heat-induced reproductive impairments [31,66]. The pregnant rats were divided into a control group, which received standard feeding, and three treatment groups (T1, T2, and T3). T1 received bee bread (0.5 g/kg body weight/day), T2 was exposed to heat stress at 43 °C, and T3 received bee bread (0.5 g/kg body weight/day) with heat stress at 43 °C, using an egg incubator for 45 min per day until delivery. Findings revealed that rats in the T2 group exhibited significant deterioration in reproductive outcomes, including decreased litter size and foetal birth weight, as well as an increased percentage of resorption and a longer gestation period compared to the control and T1 groups. In contrast, the T3 group showed improvement in these parameters, with positive effects of bee bread during heat stress exposure on litter size, foetal birth weight, and percentage of resorption indicating that bee bread supplementation attenuated the detrimental effects of heat stress on pregnancy outcomes (Nor et al. 2021) [31].

Supplementation with bee bread from stingless bee (*H. itama*) during heat stress also resulted in improved adrenal zona fasciculata thickness and reduced corticosterone levels in the experimental rats suggesting a downregulation of stress-induced hypothalamic–pituitary–adrenal axis activation [66,105]. These findings suggest the potential of bee bread as a functional dietary intervention for safeguarding against stress-induced reproductive dysfunction; nevertheless, further research, especially in humans, is necessary to validate its translational significance.

Similarly, bee bread supplementation had a positive effect on female reproductive performance by increasing the gestation period, number of pups, and duration of the oestrous cycle in 24 Sprague Dawley female rats in the study by [35]. In this study, rats were randomly divided into four treatment groups (*n* = 6): Control (0 g bee bread/kg), Treatment 1 (1 g bee bread/kg), Treatment 2 (2 g bee bread/kg), and Treatment 3 (3 g bee bread/kg). Bee bread was administered for 28 days by force feeding to ensure consistent dosage across all treated groups. Supplementation with bee bread has the potential to improve pregnancy outcomes and is effective in female reproduction [35]. These effects may be due to the fact that bee bread is rich in nutrients such as amino acids, vitamins, antioxidants, and bioactive substances that help regulate hormones and maintain proper ovarian function [106,107]. Nevertheless, further mechanistic studies and clinical investigations are essential to corroborate these findings and determine effective dosing regimens for potential therapeutic uses.

#### 4.2.2. Ovarian Function and Metabolic Disorders

Apoptosis plays a central role in ovarian function, contributing to folliculogenesis, oogenesis, oocyte selection, and follicular atresia. The balance between pro-survival and pro-apoptotic factors regulates ovarian apoptosis, ultimately determining the fate of follicular cells [108,109]. Throughout a female’s reproductive lifespan, follicles are continuously lost through apoptosis, with no mechanism to replenish the original reserve. Hormonal factors regulate this process, either promoting or inhibiting cell death, as seen in the control of turnover in various endocrine tissues [110]. At puberty, the human ovary contains approximately 400,000 follicles, yet only about 400 are ovulated during the reproductive period, meaning that over 99% undergo degeneration or atresia before menopause [110,111]. Morphological and biochemical studies have shown that apoptosis mediates the demise of both somatic and germ cells in the ovary and occurs at all stages of follicular development [112].

Bee bread from stingless bees has been reported to act as a protective agent against ovarian dysfunction and obesity induced by an HFD. Ezzat et al. (2025) studied 38 Wistar albino female rats, dividing them into a control group (*n* = 7) that received a normal diet for 14 weeks, and an HFD group supplemented with 40% HFD for 10 weeks [32]. The HFD group was further divided into four subgroups: HFD only, HFD with *S. officinalis* (300 mg/kg), HFD with bee bread (0.5 g/kg), and a normal diet for an additional 4 weeks. This design enabled comparative evaluation of therapeutic interventions and dietary reversal. In HFD rats, an irregular oestrous cycle, altered folliculogenesis and reproductive hormones, and increased body weight were observed, indicating marked reproductive and metabolic disturbances. At the molecular level, upregulation of NF-κB, TNF-α, IL-6, and caspase-3 expression, along with downregulation of CCDN1, Atg5, and PPAR, was found in the HFD group, indicating association with impaired cellular homeostasis and ovarian function [113]. In this study, supplementation with bee bread improved ovarian dysfunction in HFD rats by inducing autophagy and steroidogenic genes, and by inhibiting inflammation and apoptosis. Bee bread was shown to increase cellular clearance and protective survival mechanisms, associated with promoting Atg5-dependent autophagy, which actively blocks the intrinsic apoptotic pathway and inhibits reproductive cell death, as indicated by the suppression of Bax, p53, and caspases.

**Figure 2:**
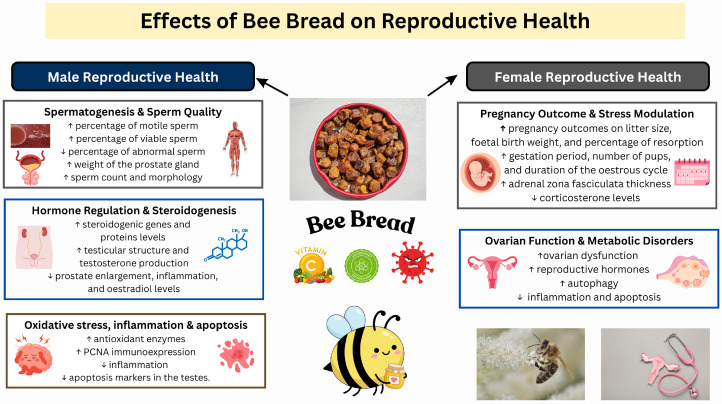
Summary of the effects of bee bread on reproductive health in males and females. Bee bread enhances male reproductive function by improving spermatogenesis, sperm quality, hormone regulation, and by reducing oxidative stress, inflammation, and apoptosis. In females, it supports reproductive outcomes by improving pregnancy parameters, regulating ovarian function and reproductive hormones, and reducing inflammation and metabolic disturbances. ↑ indicates increase; ↓ indicates decrease.

The outcomes suggest that bee bread fulfils its protective function through multiple mechanisms, including metabolic control, anti-inflammatory actions, and cellular repair processes [32,114]. This study provides evidence that bee bread supplementation can mitigate ovarian dysfunction associated with high-fat diet-induced obesity, highlighting its potential as a functional nutritional intervention for metabolic and reproductive problems. However, further research is needed to clarify its molecular mechanisms and confirm its relevance in human populations. Table 3 provides an overview of the biological activities and underlying molecular mechanisms supporting the potential benefits of bee bread in female reproductive health. Meanwhile, Figure 2 summarises the effects of bee bread on male and female reproductive health.

## 5. Safety, Dosage, and Toxicological Considerations

Bee bread from stingless bees is widely regarded as a functional food with a generally good safety profile. This is mainly attributed to its rich content of phenolic compounds, flavonoids, amino acids, organic acids, enzymes, and beneficial microbiota from fermentation [7]. Available in vitro and in vivo studies reveal low acute toxicity and no side effects at nutritionally relevant dosages, supporting its traditional use in several tropical regions, including Malaysia, Brazil, and Indonesia. However, despite these promising findings, there is a lack of consistent clinical safety evidence in humans, which has hindered its transition to clinical application [106]. Moreover, the current literature on bee bread and reproductive outcomes is largely dominated by studies reporting beneficial effects, while studies demonstrating null or adverse reproductive outcomes are not clearly reported. This may reflect a limitation in the available evidence base and potential publication bias.

There are no standardised or widely recognised clinical guidelines for human consumption of stingless bee bread regarding dosage [115]. This gap reflects a broader issue in clinical research. Most existing studies on bee bread are at the in vitro or in vivo stage, with limited evidence in humans [116]. In addition, the composition of bee bread varies greatly depending on botanical origin, geographical region, and bee species, further complicating the development of standard dosage guidelines [7,13]. Therefore, a cautious approach is advisable until more clinical data become available to define safe and effective dosage recommendations. Given these limitations, a precautionary approach is recommended. Although bee bread is traditionally consumed in moderate amounts, there is currently no clinical evidence to define a safe or effective dosage; therefore, no dosing recommendations can be established. Future research should prioritise well-controlled human trials to determine optimal dosage ranges, assess long-term safety, and standardise formulations of stingless bee bread for therapeutic and nutritional applications.

Toxicological concerns mainly relate to possible contamination from pesticide residues, heavy metals (such as lead and cadmium), and other environmental pollutants, which may vary depending on the hive’s geographical origin and surrounding vegetation.

Apiculture products such as bee bread, honey, bee pollen, royal jelly, propolis, and beeswax are widely consumed for their nutritional and therapeutic potential. Bees obtain protein and other essential nutrients from pollen, which they form into pellets by mixing it with nectar and glandular secretions. Pyrrolizidine alkaloids (PAs) are a diverse group of chemicals found in approximately 3% of all flowering plants worldwide. They are commonly present in plants of the genera Echium (Boraginaceae), Crotalaria (Fabaceae), Senecio (Asteraceae), and Eupatorium (Asteraceae), as well as in species of Argyreia, Muscari, Scilla, Cremastra, Liparis, Amphorogyne, and Osyris [117,118]. Higher concentrations of PAs are found in flowers and seeds than in leaves, stamens, and roots. PAs are considered more toxic when the necine base is unsaturated at the 1,2-position and esterified with a branched necic acid [119]. Exposure to PAs can cause both acute and chronic toxicity. Acute poisoning from PAs is mainly associated with liver damage [120], while chronic exposure has been linked to genotoxic, carcinogenic, and mutagenic effects in humans. However, there is no reported allergenic potential, and a medical case study has been published on bee bread consumption, although it may contain allergens from pollen grains and bee saliva. The allergenic potential of bee bread may be reduced by lactic acid fermentation [120]. Bee bread supplementation does not cause negative effects on the male reproductive system of rats [121].

The spontaneous lactic acid fermentation in bee bread often enhances its microbiological safety by inhibiting the growth of harmful microorganisms [122]. Improper handling or storage can promote the spread of undesirable microbes and compromise product quality. Additionally, bee bread contains allergenic proteins from pollen and bee secretions, which may pose a risk to individuals with known allergies to pollen or other apiarian products, potentially triggering allergic reactions [123]. In summary, these findings indicate that bee bread from stingless bees has significant potential as a functional food and nutraceutical; however, there is a clear need for carefully designed human clinical trials and standardised toxicological assessments to determine safe dosage ranges, long-term safety profiles, and appropriate regulatory quality control standards.

## 6. Research Gaps and Future Directions

Research on bee bread and reproductive health still has several gaps. Human clinical evidence is limited, as most available data come from preclinical studies. Well-designed randomised controlled trials are needed to evaluate its efficacy, optimal dosage, safety profile, and long-term effects on reproductive outcomes in both males and females. Although animal models provide valuable insights into potential mechanisms and therapeutic effects, their findings may not be directly translatable to humans due to species-specific differences in reproductive physiology, endocrine regulation, metabolism, and responses to bioactive compounds. Further investigation of molecular mechanisms, including gene expression, proteomics, and hormonal receptor activity, is needed to clarify its biological actions. Bee bread shows potential as a complementary therapeutic agent for managing conditions such as infertility and oxidative stress-related reproductive disorders. However, its clinical potential requires further validation. In addition, the limited number of in vitro studies investigating the cellular and molecular mechanisms of bee bread highlights the need for more mechanistic research. Cellular models, including reproductive cell lines and cultures of testicular, ovarian, granulosa, Sertoli, Leydig, and germ cells, could provide mechanistic approaches that would contribute to the study of bee bread and reproductive health.

Standardisation of bee bread composition and extraction protocols is necessary to ensure consistency across studies, as well as deeper investigation into the specific signalling pathways involved in its effects. Antioxidant and anti-inflammatory properties have been suggested, but insights into specific pathways such as oxidative stress modulation, mitochondrial function, apoptosis regulation, and endocrine signalling remain limited. Advanced approaches, including transcriptomics, proteomics, metabolomics, and epigenetic analyses, could provide a more comprehensive understanding of its biological actions. Future research should also consider population-specific responses, including differences based on age, sex, hormonal status, and underlying reproductive disorders. Interdisciplinary collaboration in reproductive biology, nutrition science, and pharmacology is essential to advance the clinical applicability of bee bread. Establishing clear regulatory frameworks and investigating synergistic effects with existing fertility treatments or antioxidants may further enhance its therapeutic potential.

Moreover, advances in cellular co-culture systems and microfluidic organ-on-a-chip platforms should be prioritised in future research to improve mechanistic understanding of bee bread in reproductive health. These systems could contribute to the reduction of animal use in accordance with the 3Rs (Replacement, Reduction, and Refinement). Such systems are believed to enable the integration of multiple reproductive cell types within a controlled microenvironment, allowing for more physiologically relevant assessment of steroidogenesis, oxidative stress regulation, endocrine signalling, and inflammatory responses. Importantly, they will also provide more precise and reproducible mechanistic data to support future clinical translation.

## 7. Conclusions

Bee bread from stingless bees shows promising effects on reproductive function through its antioxidant properties, support of spermatogenesis and steroidogenesis, improvement of pregnancy outcomes, enhancement of ovarian function, regulation of metabolism, and modulation of inflammatory activity. Findings from animal studies indicate potential benefits for both male and female reproductive health. Therefore, bee bread may become a valuable natural supplement for reproductive health management. However, well-designed randomised controlled trials are needed to evaluate its efficacy, optimal dosage, safety profile, and long-term effects on reproductive outcomes in both males and females.

## Figures and Tables

**Table 1:** Nutritional and physicochemical properties of bee bread from various stingless bee species.

Physiochemical Properties	Component	Content	Bee Species	References
Physical	Taste	Sweet	*Tetragonula angustula*, *Ptilotrigona Frieseomelitta doederlini*, and *Frieseomelitta varia*	[36]
		Bitter	*Melipona* and *Scaptotrigona*	
Chemical	Total Carbohydrate (%)	55.1 32.74–58.73 58.2 22.36	*Heterotrigona itama*	[13,42,43]
	Total Lipid (%)	2.17–4.80 5.3	*Heterotrigona itama*	[13,43]
	Crude Fibres (%)	9.30 13.65	*Meliponini seminigra* *Meliponini interrupta*	[48]
	Protein (%)	47.4 17.22–18.37 22.6	*Heterotrigona itama*	[13,42,43]
	Water Activity	0.73–0.85	*Heterotrigona itama*	[13]
	Moisture (%)	8.10 11.09–12.51	*Heterotrigona itama*	[13,42]
	Ash (%)	1.70 2.54	*Heterotrigona itama*	[13,42]
	pH	3.28	*Melipona scutellaris*	[21]

**Table 2:** Biological activities and mechanisms of bee bread in male reproductive health.

Type of Model	Treatment Dosage and Duration	Treatment Duration	Biological Activity	Mechanism of Bee Bread	Reference
12 adult male Sprague Dawley rats (8–10 weeks)	0.5 g/kg of *H. itama* bee bread	28 days	Steroidogenesis	↑ weight of the prostate gland, ↑ sperm count and morphology	[72]
32 adult male Sprague Dawley rats (10 weeks)	High-fat diet (HFD) and 0.5 g/kg of *H. itama* bee bread	12 weeks	Antioxidant Anti-inflammatory Anti-apoptotic Metabolic modulation Steroidogenesis	↓ leptin level, ↑ adiponectin level, enhanced sperm parameters and ↓ sperm DNA fragmentation, ↑ steroidogenic genes and protein levels in HFD-induced obese male rats.	[63]
24 adult male Sprague-Dawley rats	Treatment 1 (T1: 1 g of *H. itama* bee bread/kg body weight), Treatment 2 (T2: 2 g of *H. itama* bee bread/kg body weight), Treatment 3 (T3: 3 g of *H. itama* bee bread/kg body weight)	28 days	Testicular development Steroidogenesis	T2 thicker seminiferous tubular diameter, ↑ seminiferous epithelial height, ↑ testosterone level in the T1, T2 and T3 groups	[65]
48 male Kunming (KM) mice (6 weeks old)	Testosterone propionate (TP) and Bee bread from *L. flavibasis* (BBLF) group (300 mg/kg)	21 days	Anti-inflammatory Antiproliferative Steroidogenesis	TP + BBLF group exhibited significant reduction in prostate index and slightly relieved thickening and elongation of glandular epithelia, ↓ levels of oestradiol (E2), interleukin-1β (IL-1β), and interleukin-6 (IL-6) in TP + BBLF group	[33]
32 adult male Sprague Dawley rats	High-fat diet (HFD) and 0.5 g/kg of *H. itama* bee bread	12 weeks	Antioxidant Anti-inflammatory Anti-apoptotic Steroidogenesis	↑ antioxidant enzymes, ↑ PCNA immunoexpression, ↓ inflammation, ↓ apoptosis markers in the testes.	[29]
32 adult male Sprague Dawley rats (10–12 weeks old)	High-fat diet (HFD) and 0.5 g/kg of *H. itama* bee bread	12 weeks	Antioxidant Anti-inflammatory Anti-apoptotic	↑ testicular antioxidant enzymes, ↓ inflammation and apoptosis, ↑ PCNA immunoexpression, improving lactate transport	[30]

↑ indicates increase; ↓ indicates decrease.

**Table 3:** Biological activities and molecular mechanisms of bee bread in female reproductive health.

Type of Model	Treatment Dosage and Duration	Treatment Duration	Biological Activity	Mechanism	Reference
24 pregnant Sprague Dawley rats	Treatment 1: *H. itama* bee bread; 0.5 g/kg BW/day), Treatment 2: Heat stress; 43 °C of heat, Treatment 3: *H. itama* bee bread 0.5 g/kg BW/day + Heat stress; 43 °C of heat	From day 0 of pregnancy until delivery	Pregnancy outcomes during heat stress	T3 group had shown improvement as positive effects of bee bread during heat stress exposure on litter size, foetal birth weight and percentage of resorption.	[31]
24 pregnant Sprague Dawley rats (8 weeks old)	Treatment 1: 0.5 g *H. itama* bee bread/kg body weight/day, Treatment 2: standard feeding with heat exposure, Treatment 3: 0.5 g *H. itama* bee bread/kg body weight/day with heat exposure	From day 0 of pregnancy until delivery	Pregnancy outcomes during heat stress	Improvement in adrenal zona fasciculata thickness and ↓ corticosterone level.	[66]
24 mature female Sprague Dawley rats	Control: 0 g of bee bread/kg, Treatment 1: 1 g of bee bread/kg, Treatment 2: 2 g of bee bread/kg, and Treatment 3: 3 g of bee bread/kg	28 days	Pregnancy outcome	Rats in Treatment 1 ↑ gestation period compared to the Control group. The rats in Treatment 3 ↑ the number of pups and duration of estrous cycle, ↓ in percentage of resorption when compared to the Control group.	[35]
38 Wister albino female rats (4 weeks old)	HFD and bee bread (0.5 g/kg)	14 weeks	Anti-inflammatory Anti-apoptotic Metabolic modulation Autophagy activation	Bee bread improved ovarian dysfunction in HFD by inducing autophagy and steroidogenic genes and inhibiting inflammation and apoptosis.	[32]

↑ indicates increase; ↓ indicates decrease.

## Data Availability

No new data were created or analyzed in this study. Data sharing is not applicable to this article.
